# Different Types of Patient Health Information Associated With Physician Decision-making Regarding Cancer Screening Cessation for Older Adults

**DOI:** 10.1001/jamanetworkopen.2023.13367

**Published:** 2023-05-01

**Authors:** Nancy L. Schoenborn, Cynthia M. Boyd, Craig E. Pollack

**Affiliations:** Department of Medicine, Division of Geriatric Medicine and Gerontology, Johns Hopkins University School of Medicine, Baltimore, Maryland; Department of Medicine, Division of Geriatric Medicine and Gerontology, Johns Hopkins University School of Medicine, Baltimore, Maryland; Department of Health Policy and Management, Johns Hopkins University Bloomberg School of Public Health, Baltimore, Maryland

## Abstract

**IMPORTANCE:**

Although guidelines use limited life expectancy to guide physician decision-making regarding cessation of cancer screening, many physicians recommend screening for older adults with limited life expectancies. Different ways of presenting information may influence older adults’ screening decision-making; whether the same is true for physicians is unknown.

**OBJECTIVE:**

To examine how different ways of presenting patient health information are associated with physician decision-making about cancer screening cessation for older adults.

**DESIGN, SETTING, AND PARTICIPANTS:**

A national survey was mailed from April 29 to November 8, 2021, to a random sample of 1800 primary care physicians and 600 gynecologists from the American Medical Association Physician Masterfile. Primary care physicians were surveyed about breast, colorectal, or prostate cancer screenings. Gynecologists were surveyed about breast cancer screening.

**MAIN OUTCOMES AND MEASURES:**

Using vignettes of 2 older patients with limited life expectancies, 4 pieces of information about each patient were presented: (1) description of health conditions and functional status, (2) life expectancy, (3) equivalent physiological age, and (4) risk of dying from the specific cancer in the patient’s remaining lifetime. The primary outcome was which information was perceived to be the most influential in screening cessation.

**RESULTS:**

The final sample included 776 participants (adjusted response rate, 52.8%; mean age, 51.4 years [range, 27–91 years]; 402 of 775 participants were men [51.9%]; 508 of 746 participants were White [68.1%]). The 2 types of information that were most often chosen as the factors most influential in cancer screening cessation were description of the patient’s health or functional status (36.7% of vignettes [569 of 1552]) and risk of death from cancer in the patient’s remaining lifetime (34.9% of vignettes [542 of 1552]). Life expectancy was chosen as the most influential factor in 23.1% of vignettes (358 of 1552). Physiological age was the least often chosen (5.3% of vignettes [83 of 1552]) as the most influential factor. Description of patient’s health or functional status was the most influential factor among primary care physicians (estimated probability, 40.2%; 95% CI, 36.2%-44.2%), whereas risk of death from cancer was the most influential factor among gynecologists (estimated probability, 43.1%; 95% CI, 34.0%-52.1%). Life expectancy was perceived as a more influential factor in the vignette with more limited life expectancy (estimated probability, 27.9%; 95% CI, 24.5%-31.3%) and for colorectal cancer (estimated probability, 33.9%; 95% CI, 27.3%-40.5%) or prostate cancer (28.0%; 95% CI, 21.7%-34.2%) screening than for breast cancer screening (estimated probability, 14.5%; 95% CI, 10.9%-18.0%).

**CONCLUSIONS AND RELEVANCE:**

Findings from this national survey study of physicians suggest that, in addition to the patient’s health and functional status, the cancer risk in the patient’s remaining lifetime and life expectancy were the factors most associated with physician decision-making regarding cancer screening cessation; information on cancer risk in the patient’s remaining lifetime and life expectancy is not readily available during clinical encounters. Decision support tools that present a patient’s cancer risk and/or limited life expectancy may help reduce overscreening among older adults.

## Introduction

Although cancer screening has been shown to reduce cancer-related mortality and morbidity, there is increasing recognition that it can also be harmful and burdensome, especially for older adults.^1–6^ The benefits of cancer screening typically lag by 10 or more years for breast, colorectal, and prostate cancer screenings, whereas the harms and burdens of these screenings—which include complications from screening and follow-up tests, overdiagnosis and overtreatment of clinically unimportant cancers, psychological stress from false-positive results, diverted attention away from other health conditions—occur in the short term.^1–8^ Guidelines recommend against routine cancer screening for older adults for whom the harms outweigh the benefits, using age or life expectancy criteria.^5,9–18^ However, national studies show that many older adults who meet guideline criteria for cancer screening cessation continue to be screened for breast, colorectal, and prostate cancers, raising concerns for overscreening.^19–22^ Physician recommendation has been shown to be an important factor associated with overscreening among older adults, and interventions targeting clinicians to reduce overscreening are needed.^23–25^

Prior studies with older adults suggest that the type of information that is presented and how that information is framed can be important factors associated with cancer screening decisions.^26,27^ In general, older adults were receptive to tailoring cancer screening decisions based on their health and functional status but were not receptive to basing decisions on life expectancy, despite the fact that life expectancy was estimated based on the same health and functional information.^26^

To our knowledge, whether and how physicians’ decisions about cancer screening cessation are influenced by the type of patients’ health information that is presented is not known. A few prior studies have examined the association of different types of statistical information with physicians’ decision-making.^28–30^ These studies found that physicians were more likely to overestimate the benefit associated with an intervention when presented with information on relative risk reduction rather than absolute risk reduction.^29,30^ In the context of cancer screening, statistics on 5-year survival, which involves lead-time bias, were more often associated with physicians’ recommendations for screening than were statistics on reduced cancer mortality.^28,30^ Although we did not find studies that examined the association of different types of patient health information with physician decision-making, the existing literature supports that information type and framing can be significantly associated with physicians’ decisions. There are multiple ways to incorporate patient health information into screening decisions. Screening guidelines typically provide recommendations based on patient life expectancy, which can be derived from validated tools that incorporate age, health conditions, and functional status information.^5,12–18,31,32^ Another concept is that of the physiological age, sometimes also called functional age or health-adjusted age, which incorporates one’s health conditions and functional status to convey how one’s health may better match those of a different chronological age.^33–36^ Specific to the context of cancer screening, prior work has combined life expectancy and cancer-specific mortality to estimate the risk of dying from cancer in one’s remaining lifetime.^1^ Although these different types of information are derived from the same objective data (ie, an individual’s specific health conditions and functional status), it is not known whether these different types of information are associated with physician decision-making.

We aimed to examine, in a nationally representative sample, how different ways of presenting patient health information may be associated with physician decision-making regarding cancer screening cessation for older adults. Determining the appropriate type and framing of patient health information is needed to maximize the impact of decision support tools or other interventions designed to reduce overscreening.

## Methods

### Study Design

In a national cross-sectional survey, we assessed physician decision-making about cessation of screening for breast, colorectal, and prostate cancers for adults 65 years or older. We used the American Medical Association (AMA) Physician Masterfile, which contains information on all US practicing physicians, not only AMA members.^37^ We obtained a random sample of 1800 physicians in internal medicine, family medicine, general practice, and geriatric medicine (hereafter, *primary care physicians*) and 600 gynecologists because some women receive breast cancer screening through their gynecologist rather than their primary care physician. Primary care physicians were randomized to receive 1 of 3 survey versions on breast, colorectal, or prostate cancer screenings. Gynecologists were surveyed only about breast cancer screening. Sample size was informed by other national physician surveys because the study objective was exploratory.^38–40^ This study was approved by the Johns Hopkins School of Medicine institutional review board. A statement at the beginning of the survey stated that completion of the survey served as consent to participate in the study. We followed the American Association for Public Opinion Research (AAPOR) reporting guideline for survey studies with screening questions.

Physicians were ineligible if they did not care for adults 65 years or older. We also excluded physicians if they did not practice in the outpatient setting because cancer screening decisions almost always occur in the outpatient setting rather than acute or urgent care setting.

### Data Collection

We mailed the surveys, with 2 follow-up mailings to nonresponders, between April 29 and November 8, 2021. An unconditional $20 incentive was included in the first mailing. A $40 gift card was offered on survey completion in the last mailing. Physicians could respond to the paper-based survey or online. Follow-up telephone calls were made to nonresponders.

### Survey Instrument

The survey instrument was developed by the study team, pilot tested with 8 primary care physicians at our institution, and iteratively revised based on feedback. This project was part of a larger survey that also asked about perspectives on electronic medical record reminders and the definition of overscreening. Here we focus on the survey module that examined the perceived influence of different types of patient health information in decision-making. Each physician received 2 patient scenarios. One described a patient with a life expectancy of 9 to 10 years and the other described a patient with a life expectancy of 4 to 5 years. In each scenario, the physician received 4 pieces of information that described the patient’s health status in different ways, with example wording shown for Ms A, a 75-year-old woman with 9 to 10 years’ life expectancy (see eAppendix in Supplement 1 for full survey).

1. Life expectancy: for Ms A, “her life expectancy is approximately 9–10 years”;
2. Description of the patient’s health conditions and functional status: we generated descriptions such that they correlated with the estimated life expectancy for a given patient age using validated prognostic indices.^31,32^ For Ms A, “she has congestive heart failure and diabetes, and she has difficulty walking several blocks”;
3. Physiological age: we used US life tables to identify the age at which the remaining life expectancy matched that of 9 to 10 years or 4 to 5 years.^41^ For Ms A, “her physiologic age is equivalent to that of an average 80-year-old”; and
4. Risk of cancer death in remainder of life: using the same approach by Walter and Covinsky,^1^ we used the US age-specific cancer mortality rate and estimated life expectancy to calculate the risk of dying from a specific cancer in remaining lifetime.^42^ For Ms A, “her risk of dying from breast cancer in the reminder of her lifetime is approximately 1%.”
  For the 2 scenarios that a physician received, patient age, sex, and type of cancer screening were the same—only the health information varied. We selected age to correspond to guidelines in which individualized decision-making is recommended (age of 75 years for breast and colorectal screening and age of 68 years for prostate cancer screening).^9–11^ For each scenario, physicians were asked to select “which information influences you the most towards stopping [breast/colorectal/prostate] cancer screening?”

The AMA Physician Masterfile provided information on physician age, sex, year of graduation, primary specialty, and mailing address. We collected information including race and ethnicity (Asian, Black, Whte, and other [ie, American Indian or Alaska Native, Native Hawaiian or Pacific Islander, or those with >1 race]), practice setting, practice size, number of hours per week seeing patients in clinic, patient panel characteristics, whether the practice tracked physicians’ cancer screening rates, and whether patients’ screening rates may have influenced physician payment.

### Statistical Analysis

Responses were summarized descriptively. We were interested in how the primary outcome—the type of health information perceived to be most influential in screening cessation—varied by 3 key factors: patient health in the vignette (ie, life expectancy of 9–10 years vs 4–5 years), cancer screening type, and physician specialty (primary care vs gynecology). In unadjusted analyses, we compared outcomes across subgroups within each factor using the χ^2^ test. We also used a multinomial logistic regression model that examined, in the same model, patient health, cancer screening type, and physician specialty, while also adjusting for physician age, sex, race and ethnicity, geographic region, practice type, practice size, whether the practice tracked cancer screening rates, whether cancer screening rates influenced payment, number of hours worked in clinic per week, and self-reported percentage of patients who were older adults. We used robust SE estimates to account for clustering of responses by participant and estimated probabilities for each factor of interest using the margins command. All *P* values were from 2-sided tests and results were deemed statistically significant at *P* < .05. All statistical analyses were performed using Stata, version 13 (StataCorp LLC).

## Results

Of the 2400 physicians to whom surveys were mailed, 520 did not receive the surveys, including 390 surveys that were undeliverable by the postal service, 98 for whom we had an incorrect address as confirmed in follow-up telephone calls, and 32 physicians who were deceased, on medical leave, or retired (eFigure in Supplement 1). Of the remaining 1880 physicians, 993 responded (response rate, 52.8%). Compared with responders, nonresponders were more likely to be younger and internal medicine physicians; nonresponse rates did not vary by sex or geographic region (eTable 1 in Supplement 1).

Among the 993 responders, we excluded 134 who reported not caring for any older adults, 40 who reported not practicing in the outpatient setting, and 43 who did not respond to the primary outcome question for at least 1 patient vignette or chose more than 1 response for the primary outcome, leaving 776 participants (mean age, 51.4 years [range, 27–91 years]; 402 of 775 participants were men [51.9%]; 508 of 746 participants were White [68.1%]) in the analytical sample. The most common specialty represented was family medicine or general practice (316 [40.7%]), followed by internal medicine (255 [32.9%]) and gynecology (190 [24.5%]) (Table 1).^43^

**Figure 1.**
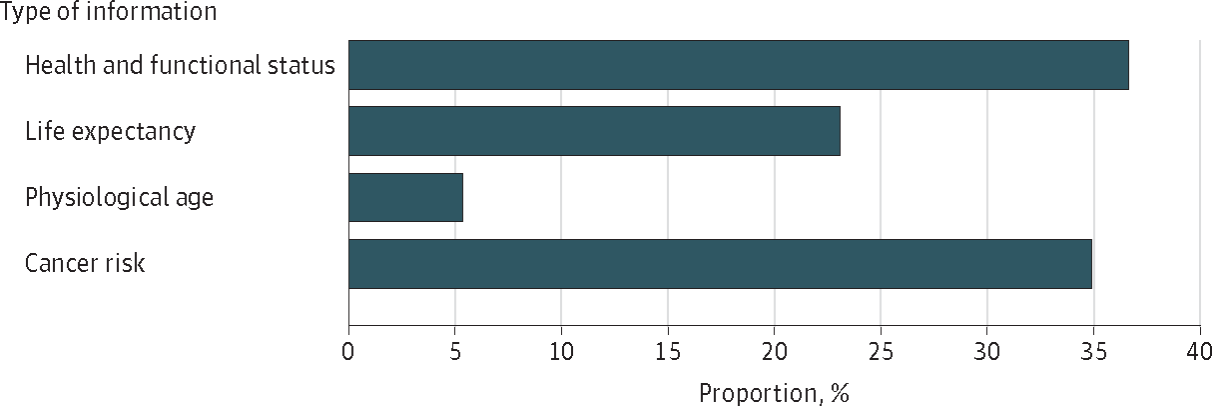
Proportion of Vignettes in Which Each Type of Information Was Chosen as the Most Influential Factor in Cancer Screening Cessation Each participant received 2 patient vignettes. One vignette described a patient with a life expectancy of 9 to 10 years, and the other described a patient with a life expectancy of 4 to 5 years. For the 776 participants, the total number of vignettes was 1552.

Across the 2 patient vignettes, description of the patient’s health conditions and functional status was most often chosen by the participants (in 36.7% of vignettes [569 of 1552]) as the most influential factor associated with cessation of screening, followed by cancer risk in the patient’s remaining lifetime (34.9% of vignettes [542 of 1552]); life expectancy was chosen in 23.1% of vignettes (358 of 1552) as the most influential factor, while physiological age was chosen in only 5.3% of vignettes (83 of 1552) as the most influential factor (Figure 1).

**Figure 2.**
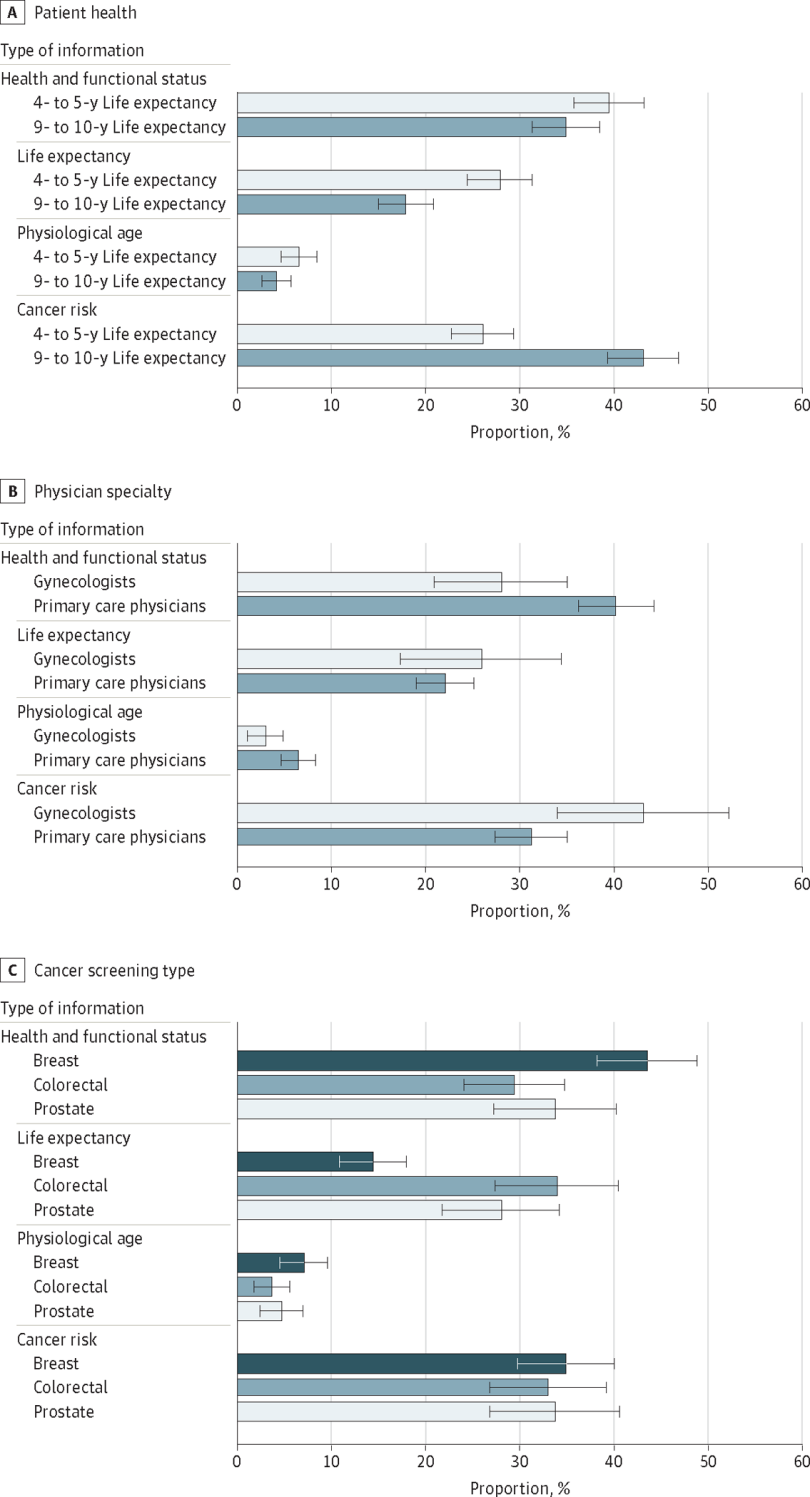
Estimated Probabilities of Physicians Choosing Each Type of Information as the Most Influential Factor in Cancer Screening Cessation Estimated probabilities were derived from a multinomial logistic regression model that included patient health, physician specialty, and cancer screening type while also adjusting for physician age, sex, race and ethnicity, geographic region, practice type, practice size, whether the practice tracked cancer screening rates, whether cancer screening rates influenced payment, number of hours worked in clinic per week, and self-reported percentage of patients who were older adults. Robust SE estimates were used to account for clustering of responses by participant. Error bars indicate 95% CIs.

In unadjusted comparisons (Table 2) and multinomial regression models (eTable 2 in Supplement 1), patient health in the vignette, physician specialty, and cancer screening type were all significantly associated with which type of information was most associated with screening cessation. When the patient had a more limited life expectancy of 4 to 5 years, health and functional status was most often chosen as the most influential factor (estimated probability, 39.5%; 95% CI, 35.8%-43.2%) followed by life expectancy (estimated probability, 27.9%; 95% CI, 24.5%-31.3%) (Figure 2A; eTable 3 in Supplement 1). When the patient had a longer life expectancy of 9 to 10 years, the risk of dying from cancer was most often chosen as the most influential factor (estimated probability, 43.1%; 95% CI, 39.3%-46.8%), followed by health and functional status (estimated probability, 34.9%; 95% CI, 31.3%-38.5%). Among primary care physicians, health and functional status was most often chosen as the most influential factor (estimated probability, 40.2%; 95% CI, 36.2%-44.2%). In contrast, gynecologists were most likely to select risk of dying from cancer as the most influential factor (estimated probability, 43.1%; 95% CI, 34.0%-52.1%) (Figure 2B; eTable 3 in Supplement 1). Finally, the perceived influence of the different types of information varied by type of cancer screening, especially for life expectancy. Physicians were more likely to choose life expectancy as the most influential for cessation of colorectal (estimated probability, 33.9%; 95% CI, 27.3%-40.5%) and prostate cancer screenings (28.0%; 95% CI, 21.7%-34.2%) compared with breast cancer screening (estimated probability, 14.5%; 95% CI, 10.9%-18.0%) (Figure 2C; eTable 3 in Supplement 1).

## Discussion

In a large, nationally representative survey of primary care physicians and gynecologists, we examined the perceived influence of different patient health information in physician decision-making about cessation of screening for 3 common types of cancers among older adults. To our knowledge, this is the first study to explore this novel topic. In addition to patient health and functional status information, we found that information about the patient’s cancer risk in their remaining lifetime (34.9%) and life expectancy (23.1%), which is information not readily available during clinical encounters, were most associated with screening cessation. Although there are multiple factors, such as patient requests, malpractice concerns, and limited visit time, associated with overscreening for cancer among older adults, our results highlight a novel consideration that can be used for interventions designed to reduce overscreening to maximize the intervention’s outcome.

Description of the patient’s health conditions and functional status was most often chosen by physicians as the most influential information when deciding about cessation of cancer screening. This is the type of information that physicians are most likely to be already familiar with about the patients. There are ongoing efforts to implement collection of functional information from older adults. For example, the Medicare annual wellness visit requires assessment of the patient’s activities of daily living and instrumental activities of daily living.^44^ The physician’s increased familiarity with this information, together with its ease of collection, underscore its likely influence in cancer screening decisions.

In contrast, a patient’s risk of dying from cancer in the remainder of their lifetime is not information that is readily available to physicians, yet this was perceived more than one-third of the time by physicians to be the most influential information when considering cancer screening cessation. A patient’s risk of dying from a specific cancer in the remainder of their lifetime is estimated by using life expectancy and population-level cancer mortality data.^1^ Guidelines increasingly recommend against routine screening for patients with limited life expectancy, but both physicians and older adults have reported skepticism about using life expectancy to guide cancer screening decisions.^26,45,46^ Reframing the discussion from limited life expectancy to low cancer risk in the patient’s remaining lifetime in clinical practice guidelines may be one way to enhance acceptability and adherence. For clinicians to use this information, however, it would likely require the implementation of decision support tools at the point of care that can present and emphasize a patient’s low risk of dying from a cancer in their remaining lifetime. One advantage of this framework of applying life expectancy to estimate individualized cancer risk is that it can be extended to other preventive care decisions, such as the risk of a cardiovascular event in a patient’s remaining lifetime when deciding about statin therapy.

We found that cancer risk information was considered more influential for gynecologists than for primary care physicians, which may reflect a difference in training and in the emphasis of professional societies’ guidelines. For example, the American College of Obstetricians and Gynecologists’ breast cancer screening guideline more systematically discusses the risk factors for breast cancer than do the guidelines from the American College of Physicians and the US Preventive Services Task Force.^11,13,47^

Physicians more often found life expectancy to be an influential factor when considering cancer screening cessation for a patient with a more limited life expectancy. In a previous study of older adults, willingness to discuss life expectancy increased when life expectancy was lower.^48^ Our finding suggests that physicians also consider life expectancy more relevant as life expectancy becomes more limited. This may be because trust in the accuracy of estimating life expectancy is higher when estimating over a shorter time horizon. It also may be that it is more straightforward to base decision-making on life expectancy when life expectancy is clearly shorter than the guideline threshold as opposed to when life expectancy is close to the threshold. Life expectancy was a more influential factor in the cessation of colorectal and prostate cancer screenings compared with breast cancer screening, even though the same life expectancy values were presented in the surveys and the guidelines use the same life expectancy threshold of less than 10 years for the cessation of routine screening across the 3 cancer types.^5,12–18^ The reason for this difference is not clear and should be explored in future studies.

Physicians chose physiological age as the most influential factor in screening decisions only 5.3% of the time. Physiological age is a common topic of study in published scientific literature. Multiple studies have developed various tools to estimate physiological age, while others emphasize its influence in myriad decisions ranging from cardiovascular treatment to transplant surgery.^33–36,49,50^ Specifically, physiological age has been used to inform individualized cancer screening recommendations for older adults.^36^ Our results suggest that practicing physicians may be unfamiliar with the term or concept of physiological age, or they may perceive it as less relevant in screening cessation decisions. Future work may seek to better understand physicians’ perceptions and understanding of physiological age and how it may influence decision-making.

### Limitations

This survey study has several limitations. It is possible that the nonresponders and those who could not have the surveys delivered may have had different attitudes and beliefs than the respondents. However, we used multiple strategies to enhance the response rate and achieved an adjusted response rate of 52.8% (776 of 1880). Second, we tested 4 common types of patient health information, but there are others we did not examine, such as risk of dying in 5 or 10 years. We also did not examine other aspects of risk communication, such as format (ie, text vs graphic displays) or framing (ie, risk of dying vs likelihood of surviving). Third, although we developed the survey questions based on literature and iterative pilot testing, the specific wording in the vignettes describing each type of health information and the hypothetical patient and the use of 2 related vignettes sequentially may have influenced results. Fourth, our study focused on the outcome of perceived influence on decision-making rather than the actual decisions; physician perceptions about what is influential in screening cessation may not match actual practice. The next step may be a randomized experiment that presents different types of health information to physicians and examines how screening recommendations may differ for patients.

## Conclusions

There are multiple ways to present patient health information; this survey study found that physicians often perceived information about the risk of dying from cancer in the patient’s remaining lifetime or life expectancy as influential factors when making decisions about cancer screening cessation for older adults. Making such information readily accessible at the point of care may help reduce overscreening.

## Supplementary Material

Supplement File 2 Data Sharing Statement

Supplement File 1 **eFigure.** Study Flow Diagram
**eTable 1.** Responder and Non-responder Characteristics
**eTable 2.** Multinomial Logistic Regression Results on Association Between Which Information Was Most Influential Towards Stopping Screening and Patient and Physician Characteristics
**eTable 3.** Predicted Probabilities of Participants Selecting Each Type of Information as Most Influential Towards Stopping Screening by Patient Health, Physician Specialty, and Cancer Screening Type
eAppendix.

## Figures and Tables

**Table 1.** Participant Characteristics and Attitude Toward Using Life Expectancy to Guide Cancer Screening in Older Adults

Characteristic	Participants, No. (%) (N = 776)^a^
Age, mean (range), y (n = 774)	51.4 (27–91)
Sex (n = 775)	
Female	373 (48.1)
Male	402 (51.9)
Race and ethnicity (n = 746)	
Asian	132 (17.7)
Black	45 (6.0)
Other^b^	61 (8.2)
White	508 (68.1)
Geographic region (n = 775)^c^	
Northeast	152 (19.6)
Midwest	190 (24.5)
South	248 (32.0)
West	185 (23.9)
Self-reported % of patient panel who are ≥65 y (n = 709)	
≤25%	268 (37.8)
>25% to 50%	247 (34.8)
>50%	194 (27.4)
No. of hours in clinic/wk, mean (range) (n = 719)	31.3 (1–100)
Specialty (n = 776)	
Family medicine or general practice	316 (40.7)
Internal medicine	255 (32.9)
Geriatric medicine	15 (1.9)
Gynecology	190 (24.5)
Practice type (n = 745)^d^	
Physician-owned practice	249 (33.4)
Health maintenance organization	62 (8.3)
Medical school or university	100 (13.4)
Nongovernment health system	239 (32.1)
Government	60 (8.1)
Other	81 (10.9)
No. of physicians in practice (n = 743)	
1	106 (14.3)
2–10	343 (46.2)
11–49	160 (21.5)
≥50	134 (18.0)
Practice tracks cancer screening rates (n = 756)	
Yes	420 (55.6)
No	336 (44.4)
Cancer screening rates associated with payment (n = 756)	
Yes	184 (24.3)
No	572 (75.7)
Type of cancer screening asked in survey (n = 776)^e^	
Breast	376 (48.5)
Colorectal	204 (26.3)
Prostate	196 (25.3)

a Totals do not always add up to 776 due to incomplete or missing data.

b Included American Indian or Alaska Native, Native Hawaiian or Pacific Islander, or those with more than 1 race.

c Geographic regions were defined based on participant’s mailing address according to the US Census Bureau region.^43^

d Participants could choose more than 1 practice type; therefore, the percentages do not sum to 100%.

e Primary care physicians were randomized to receive 1 of 3 survey versions on breast, colorectal, or prostate cancer screenings. Gynecologists were surveyed only about breast cancer screening.

**Table 2.** Number and Proportion of Vignettes in Which Each Type of Information Was Selected as Most Influential in Cancer Screening Cessation, Stratified by Patient Health, Physician Specialty, and Cancer Screening Type^a^

Stratification category	Vignettes, No. (%)				P value^b^
	Health and functional status	Life expectancy	Physiological age	Cancer risk	
Patient health					
Life expectancy 4–5 y (n = 776)	303 (39.1)	213 (27.5)	52 (6.7)	208 (26.8)	<.001
Life expectancy 9–10 y (n = 776)	266 (34.3)	145 (18.7)	31 (4.0)	334 (43.0)	
Physician specialty					
Primary care physicians (n = 1172)	441 (37.6)	298 (25.4)	67 (5.7)	366 (31.2)	<.001
Gynecologists (n = 380)	128 (33.7)	60 (15.8)	16 (4.2)	176 (46.3)	
Cancer screening type					
Breast (n = 752)	298 (39.6)	116 (15.4)	42 (5.6)	296 (39.4)	
Colorectal (n = 408)	135 (33.1)	127 (31.1)	20 (4.9)	126 (30.9)	<.001
Prostate (n = 392)	136 (34.7)	115 (29.3)	21 (5.4)	120 (30.6)	

a Each participant received 2 patient vignettes. One described a patient with a life expectancy of 9 to 10 years, and the other described a patient with a life expectancy of 4 to 5 years. For the 776 participants, the total number of vignettes was 1552.

b Comparisons across subgroups used the χ2 test.

## Data Availability

See Supplement 2.

## Key Points

### Question

How are different types of patient health information associated with physician decision-making about cancer screening cessation for older adults?

### Findings

In this national survey study of 776 physicians, description of the patient’s health conditions and functional status was most often chosen (37%) as the most influential factor associated with cessation of screening, followed by information on cancer risk in the patient’s remaining lifetime (35%) and life expectancy (23%); physiological age was chosen only 5% of the time as the most influential factor.

### Meaning

This study suggests that cancer risk in a patient’s remaining lifetime and life expectancy were among the most influential factors in physician decision-making regarding screening cessation; this type of information may be incorporated in the decision support tools to help reduce overscreening.
