# Supplementary material for: Different Types of Patient Health Information Associated With Physician Decision-making Regarding Cancer Screening Cessation for Older Adults
Source: JAMA Netw Open. Author manuscript; Available in PMC 2024 Mar 13. (PMC10935585; doi:10.1001/jamanetworkopen.2023.13367)
Supplement: Supplement File 2 — Data Sharing Statement [file NIHMS1966266-supplement-Supplement_File_2.pdf]

Data Sharing Statement

Schoenborn. Different Types of Patient Health Information Associated With Physician Decision-making Regarding Cancer Screening Cessation for Older Adults. *JAMA Netw Open*. Published May 15, 2023. doi:10.1001/jamanetworkopen.2023.13367

Data
Data available: No

Additional Information
Explanation for why data not available: This was not included in the informed consent for the study. However, we would be open to provide summary data upon request.
