# Supplementary material for: Different Types of Patient Health Information Associated With Physician Decision-making Regarding Cancer Screening Cessation for Older Adults
Source: JAMA Netw Open. Author manuscript; Available in PMC 2024 Mar 13. (PMC10935585; doi:10.1001/jamanetworkopen.2023.13367)
Supplement: Supplement File 1 — eFigure. Study Flow Diagram eTable 1. Responder and Non-responder Characteristics eTable 2. Multinomial Logistic Regression Results on Association Between Which Information Was Most Influential Towards Stopping Screening and Patient and Physician Characteristics eTable 3. Predicted Probabilities of Participants Selecting Each Type of Information as Most Influential Towards Stopping Screening by Patient Health, Physician Specialty, and Cancer Screening Type eAppendix. [file NIHMS1966266-supplement-Supplement_File_1.pdf]

Supplementary Online Content

Schoenborn NL, Boyd CM, Pollack CE. Different types of patient health information associated with physician decision-making regarding cancer screening cessation for older adults. *JAMA Netw Open.* 2023;6(5):e2313367. doi:10.1001/jamanetworkopen.2023.13367

eFigure. Study Flow Diagram

eTable 1. Responder and Non-responder Characteristics

eTable 2. Multinomial Logistic Regression Results on Association Between Which Information Was Most Influential Towards Stopping Screening and Patient and Physician Characteristics

eTable 3. Predicted Probabilities of Participants Selecting Each Type of Information as Most Influential Towards Stopping Screening by Patient Health, Physician Specialty, and Cancer Screening Type

eAppendix.

This supplementary material has been provided by the authors to give readers additional information about their work.

© 2023 Schoenborn NL et al. *JAMA Network Open.*

eFigure. Study Flow Diagram

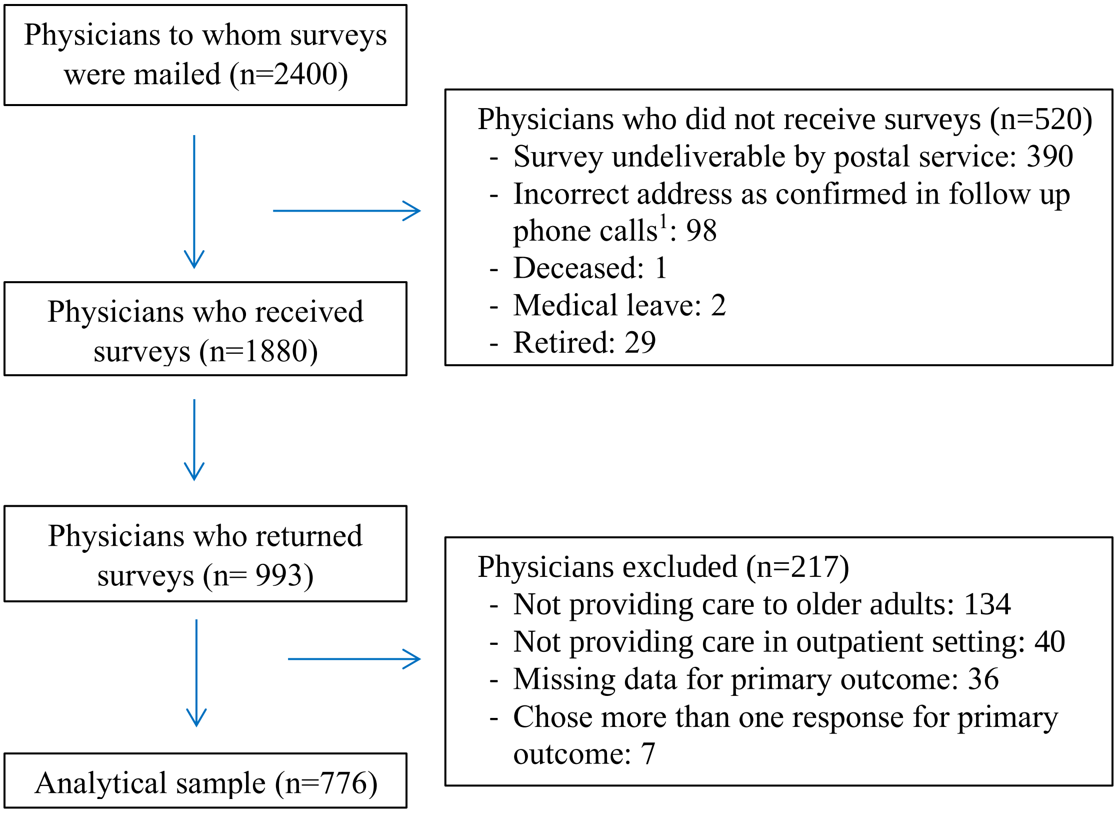

[1] Follow up phone calls were attempted for 925 non-responders who did not opt out of the study and for whom we could identify phone numbers. Of these, we could not reach 367 of them due to incorrect phone numbers, not able to reach anyone at the listed phone numbers, not able to leave messages, or if up to two messages were not returned. Of those we were able to reach, we were able to identify incorrect addresses and physicians who have been deceased, on medical leave, or retired as described. Of note, this flow diagram is slightly different than that for another survey module in the larger survey[45] as we have received two additional responses and some additional returned phone calls since the report of the other module.

© 2023 Schoenborn NL et al. *JAMA Network Open.*

eTable 1. Responder and Non-responder Characteristics

	Responders, No. (%)[1]			Non-responders No. (%), n=889	P-value comparing all responders to non-responders	P-value comparing 3 groups: respondents in the study, respondents not in the study, non-responders
	Overall n=991	Included in study (n=775) [1]	Not included in study [1,2] (n=216)			
Age, mean (SD)	52.3 (13.3)	51.4 (12.8)	55.6 (14.5)	48.8 (12.7)	<0.001	<0.001
Female	469 (47.3)	373 (48.1)	96 (44.4)	421 (47.4)	0.99	0.63
Geographic region [3]						
Northeast	207 (20.9)	152 (19.6)	155(25.5)	188 (21.2)	0.07	0.02
Midwest	225 (22.7)	190 (24.5)	35 (16.2)	170 (19.1)		
South	319 (32.2)	248 (32.0)	71 (32.9)	332 (37.4)		
West	240 (24.2)	185 (23.9)	55 (25.5)	199 (22.4)		
Specialty						
FM/GP [4]	369 (37.2)	316 (40.8)	53(24.5)	278 (31.3)	0.01	<0.001
Internal medicine	350 (35.3)	255 (32.9)	95 (44.0)	375 (42.2)		
Geriatric medicine	19 (1.9)	15 (1.9)	4 (1.9)	21 (2.4)		
Gynecology	253 (25.5)	189 (24.4)	64 (29.6)	215 (23.2)		

[1] Two respondents' surveys were missing the tracking IDs; therefore, we were not able to link them to the demographic information from the AMA.

[2] Respondents were excluded either for not meeting eligibility criteria or for missing primary outcome data (Supplemental Figure 1).

[3] Geographic regions were defined based on participant's mailing address according to the U.S. Census Bureau Region.[50]

[4] FM = family medicine; GP= general practice

© 2023 Schoenborn NL et al. *JAMA Network Open.*

eTable 2. Multinomial Logistic Regression Results on Association Between Which Information Was Most Influential Towards Stopping Screening and Patient and Physician Characteristics.[1] Reference group = description of health/functional status. Significant associations are highlighted in bold.

Participant characteristics	Life expectancy		Physiologic Age		Cancer Risk	
	RRR*	p-value	RRR	p-value	RRR	p-value
Patient in poorer health, i.e. LE 4-5 years (ref =LE 9-10 years)	**1.42**	**0.003**	1.39	0.18	**0.52**	**<0.001**
PCP (ref=gynecology)	0.57	0.08	1.51	0.32	**0.49**	**0.007**
Cancer screening type (ref=breast)						
Colorectal	**3.69**	**<0.001**	0.75	0.43	1.41	0.13
Prostate	**2.61**	**<0.001**	0.84	0.64	1.25	0.36

RRR= Relative Risk Ratio

[1] Multinomial logistic regression model also adjusted for physician age, gender, race, geographic region, practice type, practice size, whether practice tracked cancer screening rates, whether cancer screening rates affected payment, number of hours worked in clinic per week, and self-reported percentage of patient panel who are older adults. We used robust standard error estimates to account for clustering of responses by participant.

© 2023 Schoenborn NL et al. *JAMA Network Open.*

eTable 3. Predicted Probabilities of Participants Selecting Each Type of Information as Most Influential Towards Stopping Screening by Patient Health, Physician Specialty, and Cancer Screening Type.[1] Significant differences are highlighted in bold.

Covariates	Predicted probabilities in % (95% confidence intervals)			
	Health/function	Life expectancy	Physiologic Age	Cancer Risk
Patient health				
LE 4-5 years	39.5 (35.8-43.2)	**27.9 (24.5-31.3)**	6.6 (4.7-8.4)	**26.1 (22.8-29.4)**
LE 9-10 years	34.9 (31.3-38.5)	**17.9 (15.0-20.8)**	4.2 (2.7-5.7)	**43.1 (39.3-46.8)**
Physician specialty				
PCPs	**40.2 (36.2-44.2)**	22.1 (19.1-25.2)	6.5 (4.6-8.3)	31.2 (27.4-35.0)
Gynecologists	**28.0 (20.9-35.1)**	25.9 (17.3-34.4)	3.0 (1.1-4.9)	43.1 (34.0-52.1)
Cancer screening				
Breast	**43.5 (38.2-48.8)**	**14.5 (10.9-18.0)**	7.1 (4.5-9.7)	34.9 (29.8-40.1)
Colorectal	**29.4 (24.1-34.7)**	**33.9 (27.3-40.5)**	3.7 (1.8-5.5)	33.0 (26.9-39.2)
Prostate	**33.7 (27.2-40.2)**	**28.0 (21.7-34.2)**	4.7 (2.4-6.9)	33.7 (26.8-40.5)

[1] Predicted probabilities were derived from multinomial logistic regression model that also adjusted for physician age, gender, race, geographic region, practice type, practice size, whether practice tracked cancer screening rates, whether cancer screening rates affected payment, number of hours worked in clinic per week, and self-reported percentage of patient panel who are older adults. We used robust standard error estimates to account for clustering of responses by participant.

© 2023 Schoenborn NL et al. *JAMA Network Open.*

eAppendix.

SURVEY CONTENT:

Breast cancer screening version

Your opinions about stopping routine screening for breast cancer.

It is not clear whether routine breast cancer screening is beneficial after age 75. National guidelines recommend individualized decision-making in women 75 years and older. We are interested in how different information about the patient's health influence screening recommendations.

Below we present two patients. For each patient, we present 4 pieces of information about her health. Numeric estimates are made using national data.

Please assume that the patients have had regular screenings up to now**,** have no family history of breast cancer and no prior breast-related issues **-** no prior abnormal biopsies or increased genetic susceptibility to breast cancer.

Which information **influences you the most** towards stopping breast cancer screening?

Ms. A is a 75-year-old female	Choose **one**
-*She has congestive heart failure and diabetes, and she has difficulty walking several blocks.*	☐
-*Her physiologic age is equivalent to that of an average 80-year-old.*	☐
-*Her life expectancy is approximately 9-10 years.*	☐
-*Her risk of dying from breast cancer in the remainder of her lifetime is approximately 1%.*	☐

Which information **influences you the most** towards stopping breast cancer screening?

Ms. B is a 75-year-old female	Choose **one**
-*She has congestive heart failure, diabetes, and emphysema requiring oxygen. She also has difficulty walking several blocks and difficulty managing money.*	☐
-*Her physiologic age is equivalent to that of an average 90-year-old.*	☐
-*Her life expectancy is approximately 4-5 years.*	☐
-*Her risk of dying from breast cancer in the remainder of her lifetime is approximately 0.5%.*	☐

© 2023 Schoenborn NL et al. *JAMA Network Open.*

Colorectal cancer screening version:

Your opinions about stopping routine screening for colorectal cancer.

It is not clear whether routine colorectal cancer screening is beneficial after age 75. National guidelines recommend individualized decision-making in patients 75 years and older. We are interested in how different information about the patient's health influence screening recommendations.

Below we present two patients. For each patient, we present 4 pieces of information about his health. Numeric estimates are made using national data.

Please assume that the patients have had regular screenings up to now**,** have no family history of colorectal cancer and no prior colon-related issues **-** no prior adenomas or increased genetic susceptibility to colorectal cancer.

Which information **influences you the most** towards stopping colorectal cancer screening?

Mr. A is a 75-year-old male	Choose **one**
-He has diabetes and has difficulty walking several blocks.	☐
-His physiologic age is equivalent to that of an average 78-year-old.	☐
-His life expectancy is approximately 9-10 years.	☐
-His risk of dying from colorectal cancer in the remainder of his lifetime is approximately 1%.	☐

Which information **influences you the most** towards stopping colorectal cancer screening?

Mr. B is a 75-year-old male	Choose **one**
-He has congestive heart failure, diabetes, and emphysema requiring oxygen. He also has difficulty walking several blocks.	☐
-His physiologic age is equivalent to that of an average 88-year-old.	☐
-His life expectancy is approximately 4-5 years.	☐
-His risk of dying from colorectal cancer in the remainder of his lifetime is approximately 0.5%.	☐

© 2023 Schoenborn NL et al. *JAMA Network Open.*

Prostate cancer screening version:

Your opinions about stopping routine screening for prostate cancer.

National guidelines recommend individualized decision-making about prostate cancer screening for men between the ages 55-69. We are interested in how different information about the patient's health influence screening recommendations.

Below we present two patients. For each patient, we present 4 pieces of information about his health. Numeric estimates are made using national data.

Please assume that the patients have no family history of prostate cancer and no prior prostate-related issues **-** no prior abnormal screening results, abnormal biopsies or increased genetic susceptibility to prostate cancer.

Which information **influences you the most** towards stopping prostate cancer screening?

Mr. A is a 68-year-old male	Choose **one**
-He has congestive heart failure, diabetes, and has difficulty walking several blocks.	☐
-His physiologic age is equivalent to that of an average 78-year-old.	☐
-His life expectancy is approximately 9-10 years.	☐
-His risk of dying from prostate cancer in the remainder of his lifetime is approximately 0.8%.	☐

Which information **influences you the most** towards stopping prostate cancer screening?

Mr. B is a 68-year-old male	Choose **one**
-He has congestive heart failure, diabetes, and emphysema requiring oxygen. He also has difficulty walking several blocks and difficulty managing money.	☐
-His physiologic age is equivalent to that of an average 88-year-old.	☐
-His life expectancy is approximately 4-5 years.	☐
-His risk of dying from prostate cancer in the remainder of his lifetime is approximately 0.3%.	☐

© 2023 Schoenborn NL et al. *JAMA Network Open.*

Your background and practice characteristics

If you work at more than one practice, please think about the practice you work at the most.
Which of the following best describes your current employer? (*Check all that apply)*

- ☐ Physician-owned practice
- ☐ Health maintenance organization (HMO)
- ☐ Medical school or university
- ☐ Non-government medical group or health care system
- ☐ Government
- ☐ Free-standing clinic
- ☐ Other: ___________________________

Including yourself, about how many physicians work at your practice?

- ☐ 1 (solo practitioner)
- ☐ 2-10
- ☐ 11-49
- ☐ 50+

About how many hours per week do you see patients in clinic?

_____ hours / week

Does your practice track the rate of cancer screening among your patients?

- ☐ Yes
- ☐ No
- ☐ Don't know

Do your patients' cancer screening rates affect your payment in any way?

- ☐ Yes
- ☐ No
- ☐ Don't know

About what proportion of your patients are 65 years or older ______%

Are you Hispanic or Latino?

- ☐ Yes
- ☐ No

© 2023 Schoenborn NL et al. *JAMA Network Open.*

What is your race? (*Check all that apply)*

- ☐ American Indian or Alaskan Native
- ☐ Asian
- ☐ Black of African American
- ☐ Native Hawaiian or Pacific Islander
- ☐ White
- ☐ Other: ________________________

© 2023 Schoenborn NL et al. *JAMA Network Open.*
